# Lung exposure of titanium dioxide nanoparticles induces innate immune activation and long-lasting lymphocyte response in the Dark Agouti rat

**DOI:** 10.3109/1547691X.2010.546382

**Published:** 2011-02-10

**Authors:** Åsa Gustafsson, Elsa Lindstedt, Linda Svensson Elfsmark, Anders Bucht

**Affiliations:** 1 Division of CBRN Defense and Security, Swedish Defense Research Agency, Umeå, Sweden; 2 Department of Public Health and Clinical Medicine, Umeå University, Umeå, Sweden; 3 Department of Oncology, Radiology and Clinical Immunology, University Hospital, Uppsala, Sweden

**Keywords:** Nanoparticles, TiO_2_, lung, inflammation, NK cells, T-cells, dendritic cells

## Abstract

Nanomaterial of titanium dioxide (TiO_2_) is manufactured in large-scale production plants, resulting in risks for accidental high exposures of humans. Inhalation of metal oxide nanoparticles in high doses may lead to both acute and long-standing adverse effects. By using the Dark Agouti (DA) rat, a strain disposed to develop chronic inflammation following exposure to immunoactivating adjuvants, we investigated local and systemic inflammatory responses after lung exposure of nanosized TiO_2_ particles up to 90 days after intratracheal instillation. TiO_2_ induced a transient response of proinflammatory and T-cell-activating cytokines (interleukin [IL]-1α, IL-1β, IL-6, cytokine-induced neutrophil chemoattractant [CINC]-1, granulocyte-macrophage colony-stimulating factor [GM-CSF], and IL-2) in airways 1-2 days after exposure, accompanied byaninfluxofeosinophilsand neutrophils. Neutrophil numbers remained elevated for 30 days, whereas the eosinophils declined to baseline levels at Day 8, simultaneously with an increase of dendritic cells and natural killer (NK) cells. The innate immune activation was followed by a lymphocyte expansion that persisted throughout the 90-day study. Lymphocytes recruited to the lungs were predominantly CD4^+^ helper T-cells, but we also demonstrated presence of CD8^+^T-cells, B-cells, and CD25^+^T-cells. In serum, we detected both an early cytokine expression at Days 1-2 (IL-2, IL-4, IL-6, CINC-1, IL-10, and interferon-gamma [IFN-γ] and a second response at Day 16 of tumor necrosis factor-alpha (TNF-α), indicating systemic late-phase effects in addition to the local response in airways. In summary, these data demonstrate a dynamic response to TiO_2_ nanoparticles in the lungs of DA rats, beginning with an innate immune activation of eosinophils, neutrophils, dendritic cells, and NK cells, followed by a long-lasting activation of lymphocytes involved in adaptive immunity. The results have implications for the assessment of risks for adverse and persistent immune stimulation following nanoparticle exposures in sensitive populations.

## Introduction

Inhaled nanosized particles (NPs) are implicated as a contributing factor to the adverse health effects of air pollution, especially in individuals with asthma or cardiovascular disease (MacNee and Donaldson, 2003; Kelly and Sandström, 2004; Mills et al., 2007; Törnqvist et al., 2007). Although certain properties (e.g., organic content and uniformity) distinguish engineered NPs from anthropogenic NPs, there are likely common effects and mechanisms of toxicity. NP toxicity is generally described in terms of oxidative stress, inflammation, adjuvant, and procoagulant effects, and interaction with biomolecules that might lead to unwanted toxic effects in the body (Nel et al., 2006; Li et al., 2008).

Nanosized titanium dioxide (TiO_2_) has photocatalytic properties and is produced in increasing amounts for energy and environmental applications, as well as use in pigments and medical implants. Previous studies have shown that inhaled TiO_2_ particles can cause oxidative damage, induce pulmonary inflammation and emphysema, and that chronic exposure can lead to pulmonary tumors (Oberdörster et al., 1994; Warheit et al., 1997, 2006, 2007; Renwick et al., 2004; Ma-Hock et al., 2009).

Recently, it has been reported that lung exposure to nano-TiO_2_ NPs in mice cause inflammation by activation of T-helper- 2 cells (T_H_2), with a function primarily in humoral immune responses and allergic sensitization (Park et al., 2009; Larsen et al., 2010). Since species differences in the pulmonary effects of TiO_2_ have previously been reported, it is not clear whether such immune activation canbe generalized, thatis, the rat has been described to be more sensitive to TiO_2_ NPs than both the mouse and hamster (Bermudez et al., 2004). Furthermore, it is likely that gene regulation of immune responses differ between inbred strains within a species. From previous studies, inbred rat strains have shown to differ in susceptibility to various models of human immune-mediated and inflammatory diseases. The Dark Agouti (DA) rats have previously been studied in experimental autoimmune diseases such as arthritis and encephalomyelitis due to their high susceptibility to develop long-lasting immune-mediated disorders (Gasser et al., 1973; Griffiths et al., 1981; Battisto et al., 1982; Eishi and McCullagh, 1988).

In the present study, we investigated the effects of acute TiO_2_ NPs exposure thatmay occur by accidentin work environments handling large amounts of powdered TiO_2_ NPs. The dose chosen for the exposures corresponds approximately to a human exposure for 8 h at a concentration of 12mg/m^3^, taking in consideration the differences in respiratory frequency and respiratory volume between human and rats. According to Occupational Safety and Health Administration (OSHA), the occupational permissible exposure limit (PEL) is 15mg/m^3^ for TiO_2_ as total dust and 5mg/m^3^ for TiO_2_ as respirable dust (8-h time-weight average concentration) (NIOSH, 2005). However, it has been reported that the air concentrations of TiO_2_ do not generally exceed 1–5mg/m^3^ in workplaces where TiO_2_ particles are milled and packed, but higher concentrations might be accidently released.

We aimed here to: (1) establish a model of a single exposure of TiO_2_ NP in a rat strain that is highly susceptible to inflammatory disorders, representing more sensitive individuals in a population and (2) determine the time sequence of adverse immune reactions and the putative development of lung injury during a period of 90 days post-exposure.

## Materials and methods

### Animals

Inbred pathogen-free male DA rats (B&K, Sollentuna, Sweden) 10-11-weeks-old were housed in a restricted-access animal care facility. They facilities were maintained at 20-24°C, with a 50% relative humidity, and with a 12-h on/off light cycle; all ratswerepermitted access to food and water *ad libitum*. All animal experimental procedures used herein were approved by the Animal Research Ethical Committee in Umeå, Sweden.

### Particles

Nanosized TiO_2_ particles (P25; Degussa AG, Frankfurt, Germany) consisting of 75% anatase and 25% rutile were kept dark throughout the experiments, suspended in phosphate-buffered saline (PBS, pH 7.4), and ultrasonicated for 30 min prior to use. Primary particle size was 21 nm according to the manufacturer. Static light-scattering analysis after sonication (Laser Scattering Particle Size Distribution Analyzer LA-950; Horiba Instruments Inc., Södertälje, Sweden) indicated two fractions of agglomerated particles of median size 200 nm and 2 µm, respectively (data not shown).

### Nanoparticle exposure

Rats were anesthetized with 4% isoflurane (Abbot Scandinavia AB, Solna, Sweden) and intratracheally instilled once with TiO_2_ suspended in 200 µL PBS. The dose was chosen from a dose–response study where 1, 5, and 7.5 mg TiO_2_/kg were compared with exposure for vehicle only (PBS) and evaluated 24 h after exposure. The dose 5 mgTiO_2_/kg body weightwas used in a time–kinetic study where exposed animals were sacrificed at 1, 2, 8, 16, 30, and 90 days post-instillation and comparisons were performed vs. non-instilled animals (time- point zero). In order to exclude age-dependent changes, we also compared with control animals exposed for PBS only, sacrificed at 2, 16, 30, and 90 days post-instillation.

### Bronchoalveolar lavage

Rats were sacrificed by an intraperitoneal injection of sodium pentobarbiturate (Apoteket AB, Stockholm, Sweden) followed by exsanguination from the descending aorta. Bronchoalveolar lavage fluid (BALF) was collected with 5× 5 mL ice-cold Ca^2+^, Mg^+^-free Hanks' balanced salt solution (Sigma-Aldrich, St. Louis, MO) at 1, 2, 8, 16, 30, and 90 days post-instillation. Cell pellets were resuspended in PBS and cell counts were determined using manual trypan blue dye exclusion. Cells were analyzed by flow cytometry and light microscopy, whereas cell-free BALF and serum were used for measurements of secreted cytokines.

### BALF cell count

Leukocyte differential count was determined by applying replicates of 30,000 cells onto microscope slides using a Shandon Cytospin 3 (Shandon Southern products Ltd., Runcorn, UK). Slides were fixed and thereafter stained with May–Grünwald–Giemsa prior to manual cell differential count, in blinded fashion, using light microscopy to assess morphology of 300 cells/slide.

### Flow cytometry

Antibody staining was performed in 96-well plates with 2.5 × 10^5^ cells/sample (note: BAL specimens containing a lower number of cells were pooled). Flow cytometry was performed using a BD FACSort™ (Becton Dickinson, San Jose, CA) according to standard procedure and analyzed with BD FACSDiva Software. Monoclonal antibodies (and the associated fluorophore conjugate) used in this study were anti-: CD3-FITC; CD3-PE; αβ-TCR-PerCP; γδ-TCR-FITC; CD45RA-PE; NKR-P1A-PE; CD8a-PerCP; CD4-PE-Cy5; CD25-PE; IgG1-FITC; OX-62, and OX-6-PerCP; isotype controls were also employed. Non-specific binding was blocked by incubation with anti-rat CD32 (F_cγ_ II-receptor) prior to specific staining, except when dendritic cells were investigated. All antibodies were from BD Sciences Pharmingen (San Diego, CA). T-Cells were defined as CD3^+^, B-cells were defined as CD3^−^ CD45RA^+^, and natural killer (NK) cells as CD3^−^ NKR-P1A^+^. Dendritic cells were identified as CD3^−^ CD45RA^−^ OX-62^+^ OX-6^+^ as previously described by Lambrecht et al. (1999).

### Cytokine measurements

Serum- and cell-free BALF were used for cytokine analysis. Using a Luminex Bio-Plex 200 System (Bio-Rad, Hercules, CA), interleukin (IL)-1α, IL-1β, IL-2, IL-4, IL-6, IL-10, granulocyte-macrophage colony-stimulating factor (GM-CSF), interferon-gamma (IFN-γ), and tumor necrosis factor-alpha (TNF-α) levels were measured (Rat 9-Plex A Panel; Bio-Rad). Vascular endothelial growth factor (VEGF) and cytokine-induced neutrophil chemoattractant (CINC-1, a homolog of human IL-8) were quantified using ELISA kits (ELISA Duoset; R&D Diagnostics, Minneapolis, MN) according to the manufacturers' instructions; in the case of serum VEGF, K Blue Enhanced substrate (Neogen Europe Ltd., Ayr, Scotland) was used. The plates were read at 450 nm with a wavelength correction at 570 nm (Labsystems iEMS Reader MF, Vantaa, Finland). Using Thermo Electron Ascent Software, the absorbance was transformed to pg/mL, using standard curves prepared with cytokine standards included in the kits.

### Lung histopathology

At 2, 30, and 90 days post-instillation, two TiO_2_-exposed and two PBS-exposed rats were sacrificed by

exsanguination under sodium pentobarbiturate anesthesia (lethal dose). Their lungs were immediately rinsed from blood with PBS that was injected through the right ventricle. Phosphate-buffered 4% paraformaldehyde (Solveco, Chemicals AB, Stockholm) was used to inflate the lungs through airway infusion at constant pressure (20 cm H_2_O), whereupon lungs and heart were removed *en bloc* and fixed in buffered 4% paraformaldehyde. Lungs were paraffin-embedded, sectioned, stained with hematoxylin-eosin or Masson trichrome stain (Sigma-Aldrich) and evaluated by light microscopy.

### Statistical analysis

The statistical analyses of differential cell counts in BALF were performed with one-way ANOVA and Dunnett's post-hoc test. In the dose-response experiment, the TiO_2_-exposed animals were compared with a control group exposed for PBS only. In the kinetic study the TiO_2_-exposed animals, sacrificed at 1, 2, 8, 16, 30, and 90 days post-exposure, were compared with non-exposed animals. For the later timepoints 16, 30, and 90 days post-exposure, the nano-TiO_2_-exposed animals were also compared with PBS-exposed animals sacrificed at corresponding timepoints using Student's unpaired *t*-test. The statistical analyses of lymphocyte subsets by flow cytometry were performed with one-way ANOVA and Dunnett's post-test and compared with non-instilled animals for all groups. For cytokine measurements, the concentration in samples below the detection limit was set to 0, and a Kruskal–Wallis test with Dunn's post-test (two-tailed) was therefore conducted. For correlation analysis, Spearman's rank test was performed. Data analyses were considered significant at *P* < 0.05. Results are expressed as mean (±SEM) in graphs and mean (±SD) in tables.

## Results

### Nanosized TiO_2_ induces an acute airway inflammation and sustained lymphocyte response

**Figure 1:**
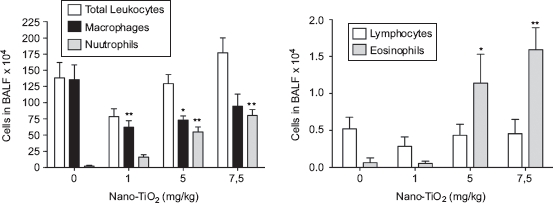
Dose-dependent differences in the number of cells in bronchoalveolar lavage fluid from rats 24h after intratracheal instillation with nanosized TiO_2_. One-way ANOVA with Dunnett's post-test; value is significantly (*P < 0.05 and **P < 0.01) different vs. phosphate-buffered saline (PBS) control. Data are presented as mean ± SEM (*n* = 6).

The number of leukocytes in the lungs 1 day after lung exposure to increasing concentrations of TiO_2_ NPs (0, 1, 5, and 7.5 mg/kg body weight) was evaluated. A dose-dependent increase in eosinophils and neutrophils, as well as a decrease in macrophages (Figure 1), was detected. For further analysis, the 5 mg/kg dose was chosen to investigate the effects at 1, 2, 8, 16, 30, and 90 days post-exposure. The dose selected was the lowest that resulted in significantly increased leukocyte response in BALF 1 day after exposure.

**Figure 2:**
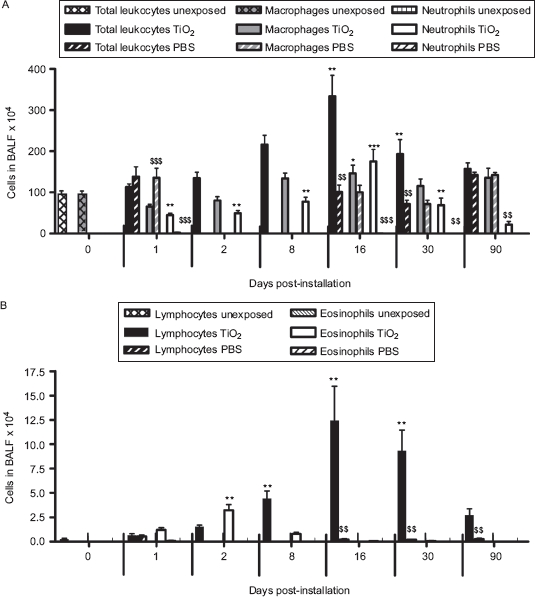
The number of cells in bronchoalveolar lavage fluid from nano-TiO_2_ (5mg/kg)-exposed rats 0 (*n* = 10), 1 (*n* = 20), 2 (*n* = 15), 8 (*n* = 11), 16 (*n* = 5), 30 (*n* = 6), and 90 (*n* = 6) days post-intratracheal instillation. One-way ANOVA with Dunnett's post hoc test was employed, and values significantly changed vs. nonexposed control animals (timepoint 0) are indicated (*P < 0.05, **P < 0.01,***P < 0.001). A Student's *t*-test was performed to compare TiO_2_-exposed rats with that of rats exposed for phosphate-buffered saline (PBS) only at Days 1, 16, 30, and 90 (^$$^*P* < 0.01, ^$$$^*P* < 0.001). Data are presented as mean ± SEM.

**Figure 3:**
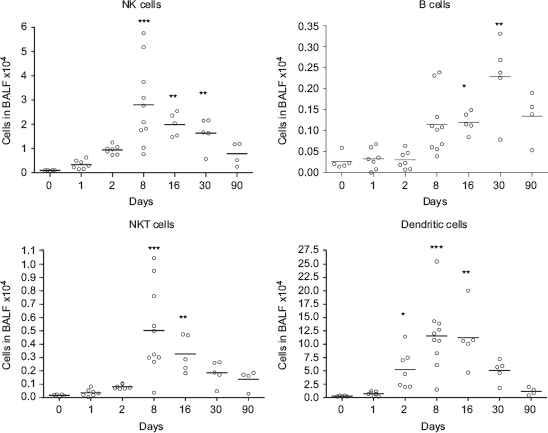
Numbers of NK T-cells (CD3^+^ NKR-P1A^+^), NK cells (CD3^+^ NKR-P1A^+^), B-cells (CD3^−^ CD45^+^), and dendritic cells (CD3^−^ CD45RA^−^ OX-62^+^ OX-6^+^) in bronchoalveolar lavage fluid from nanosized TiO_2_ (5 mg/kg) exposed rats 0 (*n* = 5), 1 (*n* = 7), 2 (*n* = 7), 8 (*n* = 10), 16 (*n* = 5), 30 (*n* = 5), and 90 (*n* = 4) days after a single intratracheal instillation. Kruskal–Wallis test with Dunn's post-test; value is significantly (*P < 0.05, **P < 0.01, ***P < 0.001) different vs. control.

**Figure 4:**
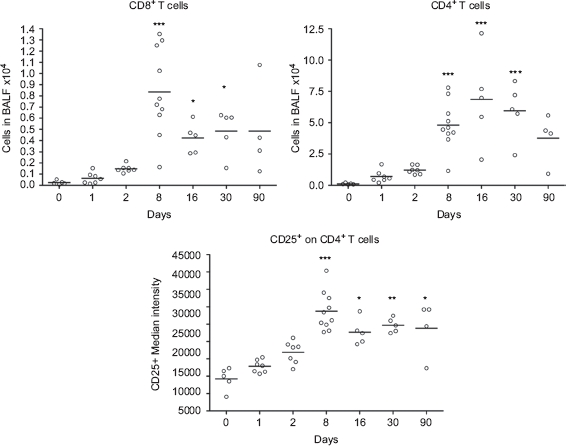
Numbers of cytotoxic T-cells (CD3^+^ CD8^+^) and CD4^+^ T-cells, and median intensity of CD25 receptor expression on CD4^+^ T-cells in bronchoalveolar lavage fluid from nanosized TiO_2_ (5 mg/kg) exposed rats 0 (*n* = 5), 1 (*n* = 7), 2 (*n* = 7), 8 (*n* = 10), 16 (*n* = 5), 30 (*n* = 5), and 90 (*n* = 4) days after a single intratracheal instillation. Kruskal–Wallis test with Dunn's post-test; value is significantly (*P < 0.05, **P < 0.01, ***P < 0.001) different vs. control.

One single instillation of 5mg nano-TiO_2_ NPs/kg induced early eosinophil and neutrophil recruitment to the airways appearing from Day 1 post-exposure when comparedwith unexposed animals (Figure 2). The eosinophils were elevated until Day 8, whereas the neutrophils remained elevated for at least 30 days. Concomitant with the neutrophilia, a transient increase of dendritic cells was detected with a peak cell numbers at Day 8 (Figure 3), followed by maximal lymphocyte cell numbers at Day 16, which persisted throughout the 90-day study (Figure 2). The nano-TiO_2_-exposed animals were also compared with animals exposed for PBS only. The numbers of neutrophils, lymphocytes, and eosinophils were not increased following PBS instillation (Figure 2) compared with baseline cell numbers in BALF of healthy animals, although a small increase of macrophages was observed at Day 90. Among the lymphocytes, NK cells and T-cells expressing the NKR-P1A receptor (NK T-cells) displayed a transient increase at the same timepoints as dendritic cells (Figure 3). NK cells recruited to the airways expressed high density of the NKR-P1A receptor on the cell surface as indicated by the increased NKR-P1A^bright^ population at Days 2, 8, and 16 (Table 1). The lymphocyte response was dominated by T-cells, including CD4^+^ helper T-cells with high expression of CD25 (CD25^bright^) (Figure 4). Smaller fractions of lymphocytes were identified as CD8^+^ cytotoxic T-cells and B-cells (Figures 3 and 4). T-Cells recruited to the airways were predominantly of the T-cell receptor (TCR) αβ subset with a minor proportion of T-cells expressing the γδ TCR (data not shown).

**Table 1:** Proportion of natural killer (NK) cells expressing NKR-P1A^bright^

Days	0 (*n* = 10)	1 (*n* = 14)	2 (*n* = 15)	8 (*n* = 11)	16 (*n* = 5)	30 (*n* = 6)
% Bright	36 ± 3	30 ± 2	72 ± 2**	70 ± 3**	62 ± 2**	43 ± 4

Expressed as mean percentage of total NK numbers ± SD.

One-way ANOVA with Dunnett's post-test; value significantly (**P < 0.01,) different as compared with control.

### Nanosized TiO_2_ induces cytokine release in BALF and serum

**Figure 5:**
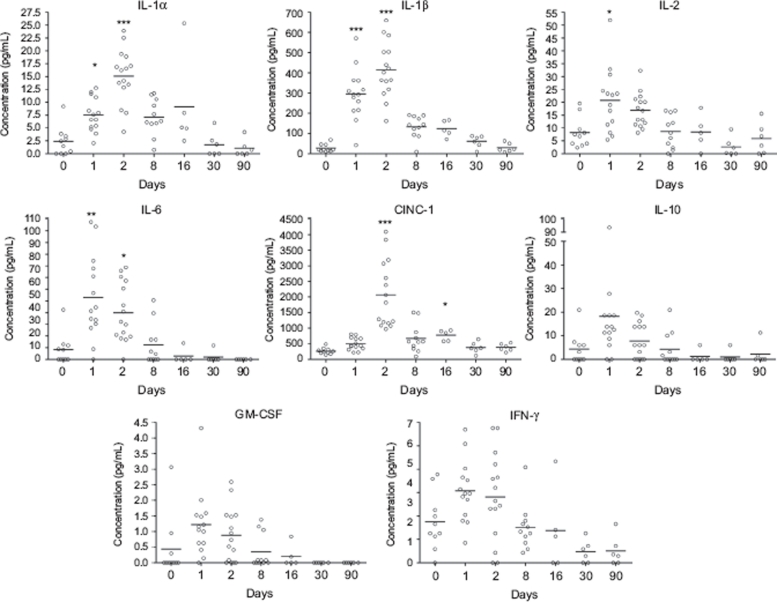
Concentration of cytokines in bronchoalveolar lavage fluid from nanosized TiO_2_ (5 mg/kg)-exposed rats 0 (*n* = 10), 1 (*n* = 14), 2 (*n* = 15), 8 (*n* = 11), 16 (*n* = 5), 30 (*n* = 6), and 90 (*n* = 6) days after a single intratracheal instillation. Kruskal–Wallis test with Dunn's post-test; value is significantly (*P < 0.05, **P < 0.01, ***P < 0.001) different vs. control.

**Figure 6:**
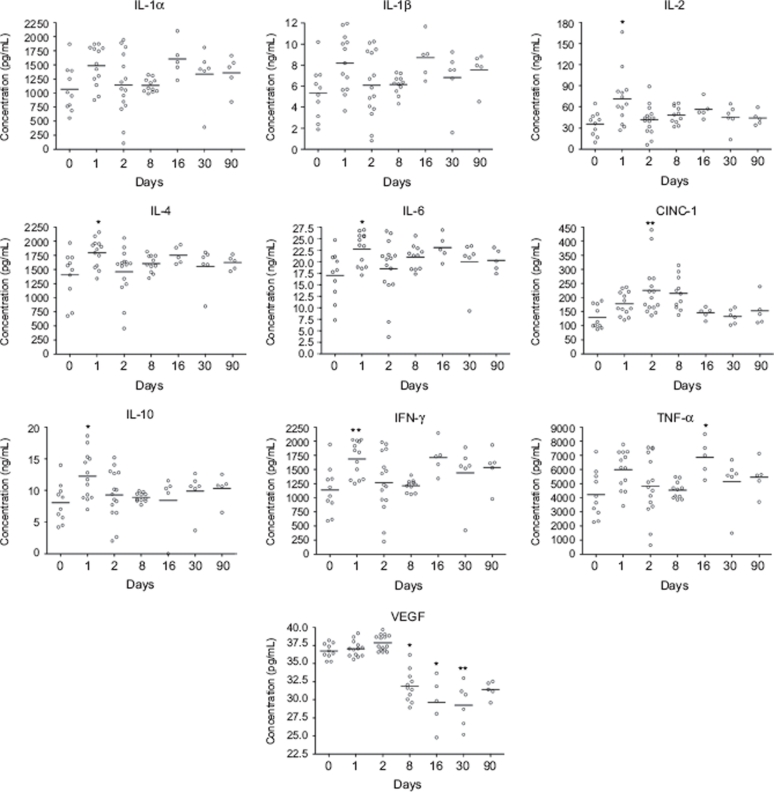
Concentration of cytokines in serum from nano-TiO_2_ (5 mg/kg) exposed rats 0 (*n* = 10), 1 (*n* = 13), 2 (*n* = 15), 8 (*n* = 11), 16 (*n* = 5), 30 (*n* = 6), and 90 (*n* = 5) days after a single intratracheal instillation. Kruskal–Wallis test with Dunn's post-test; value is significantly (*P < 0.05, **P < 0.01) different vs. control.

At Days 1–2 post-exposure, an early and transient increase of IL-1α, IL-1β, IL-2, IL-6, CINC-1, and GM-CSF was detected in BALF (Figure 5). At the same timepoint, elevated levels of IL-2, IL-4, IL-6, IL-10, and IFN-γ were detected in serum, whereas the increase in serum concentration of CINC-1 was detected at Days 2–8 post-exposure (Figure 6). A biphasic cytokine response was detected in serum at Day 16 as indicated by increased TNF-α (Figure 6). VEGF concentration in serum was decreased from Days 8 to 90 when compared with levels associated with non-exposed animals (Figure 6), although the difference from PBS-instilled controls at corresponding timepoints was not statistically significant.

### Accumulation of particles in lung epithelium and examination of lung fibrosis

**Figure 7:**
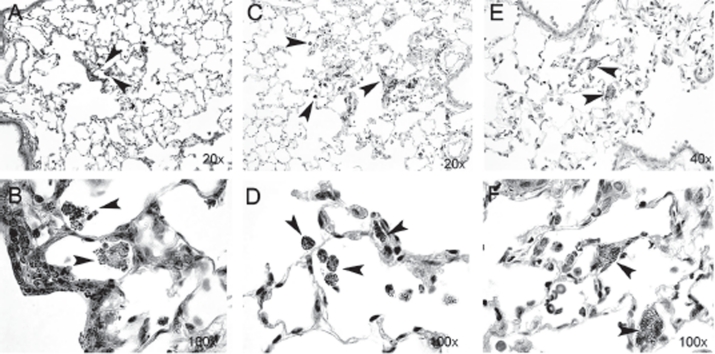
Lung sections from rat tissue showing particle uptake at Days 2 (A and B), 30 (C and D), and 90 (E and F) post-nano-TiO_2_ exposure. Particle aggregates (arrows) can be found in alveolar macrophages at Days 2 and 30, and mainly in the interstitium at Day 90. The tissues were stained with hematoxylin-eosin. (See colour version of this figure online at http://www.informahealthcare.com/imt)

Morphological examination of lung tissue sections 2 days after exposure revealed free particle aggregates in close association to terminal bronchioles and alveolar ducts. A minor uptake of particle aggregates in alveolar macrophages (AM) was observed (Figure 7). Thirty days post-exposure, the presence of particle aggregates in macrophages had increased and few free particle aggregates were seen. At Day 90, particle aggregates were predominantly found within the macrophages. The macrophages were doubled in size and contained larger amounts of vacuoles, compared with the macrophages at Day 30. We also observed cell-shaped areas of aggregates, possibly as a consequence of disrupted cells due to particle “overload” (Oberdörster et al., 1992). To evaluate possible fibrosis, tissue sections were stained for collagen deposition at Day 90 post-exposure, but histo-pathological examination revealed no lung tissue fibrosis, epithelial injury, or granuloma formation in TiO_2_-exposed animals (data not shown).

## Discussion

In the present study, a single dose of high concentration of nanosized TiO_2_ particles caused a dynamic inflammatory response in airways of DA rats, characterized by a transient influx of eosinophils and a more sustained neutrophilic response, followed by a recruitment of dendritic cells and lymphocytes expressing NK receptors (NK cells and NK T-cells). The transient innate immune response resulted in a late-phase recruitment of lymphocytes involved in adaptive immunity, predominantly CD4^+^ T-cells. We did not observe any signs of epithelial injury or lung fibrosis, indicating that the TiO_2_ dose given to the animals (5 mg/kg body weight) did not produce severe cytotoxic effects in the lung epithelium.

The innate cellular response was preceded by an increase of pro-inflammatory cytokines IL-1α, IL-1β, IL-6, CINC-1, and GM-CSF in BALF 1-2 days post-exposure. Consistent with our observations of the subsequent recruitment of inflammatory cells to the airways, the expression of these cytokines in concert provides strong signals for neutrophil chemoattraction (Nakagawa et al., 1994), activation of the Th17 pathway (Mills, 2008), prolonged survival of eosinophils in the microenvironment (Lampinen et al., 2004), as well as dendritic cell proliferation and maturation (Dieu et al., 1998). Previous studies in other rat strains have shown dose-dependent transient increases in granulocytes and monocytes in the lungs, as well as epithelial and fibroproliferative changes upon challenge with TiO_2_ (Oberdörster et al., 1994; Bermudez et al., 2004; Ahn et al., 2005; Warheit et al., 2007; Sager et al., 2008; Kobayashi et al., 2009; Ma-Hock et al., 2009). These differences in results may be due to different properties of TiO_2_, like crystal structure, particle size, surface chemistry, and surface area. By that means, it is difficult to compare results between different NP studies. Growing evidence suggests that TiO_2_ may cause different adverse health effects depending on the crystal structure and size of the particles (Warheit et al., 1997, 2006, 2007; Kobayashi et al., 2009). *In vitro* studies have shown that TiO_2_ particles of different crystal structures exert different toxic effects, for example from exposures on respiratory epithelial cells it appears that the anatase phase of nanocrystalline TiO_2_ is more toxic than the rutile phase, probably due to a high photocatalytic activity of anatase resulting in effective generation of highly reactive oxygen species (ROS) (Gurr et al., 2005; Sayes et al., 2006; Singh et al., 2007; Hussain et al., 2009). It is, however, not well understood whether this mechanism of toxicity can trigger inflammatory responses *in vivo*.

Ma-Hock and colleagues have described nano-TiO_2_ (14% rutile, 86% anatase) accumulation in lymphoid tissue upon inhalation exposure in the Wistar rat, indicating translocation of inhaled particles to the lymph nodes possibly through uptake by migratory antigen-presenting cells (Lorentzen et al., 1997; Dimitrijevic et al., 2001). The Wistar, much like the DA rat, is disposed to develop T_H_1 -mediated inflammation involving strong dendritic cell and macrophage activation, although studies have shown that the DA rat is even more prone to develop T_H_1 inflammatory disorders (Lorentzen et al., 1997; Dimitrijevic et al., 2001). Our data on expansion of dendritic cell in the lung together with the observation of time-dependent clearance of particles from the alveolar compartments further support a particle trans-location to lymphoid tissue, but further studies have been conducted to confirm this scenario. In our study, we observed that TiO_2_ particle aggregates were initially free dispersed in alveolar regions with subsequent uptake in AM. With time the macrophages increased in size containing accumulating numbers of NPs and finally the macrophages disrupted, resulting in release of particles into the lung again. This observation is consistent with AM as the first-line defense against inhalation of particles, acting by phagocytosis and degradation through intracellular processes. It is known that AM can turn into an overload state, if the internal volume of particles is greater than 60%, which inhibit their function (Oberdorster et al., 1992). It is conceivable that particle uptake followed by particle release as a consequence of macrophage disruption has an influence on the dynamic inflammatory response observed.

We demonstrated lymphocyte influx to the lungs, dominated by CD4^+^ T-cells and with smaller fractions of CD8^+^ T-cells and B-cells, indicating initiation of an adaptive immune response, although the specific antigens recognized by the cells remains to be defined. A NP introduced into a biological system may rapidly adsorb proteins forming a protein corona (Lundqvist et al., 2008) that, in turn, could constitute signals for cells. Furthermore, protein adsorption onto NPs could induce conformational changes of the adsorbed proteins, as evidenced by a study by Lundqvist et al. (2006) where silica NPs were shown to induce a helical structure, including a catalytic site, on unstructured peptides in solution. Conformal changes *in vivo* could lead to a change or loss of function of the adsorbed proteins; it may also result in presentation of novel peptide motifs to the immune system. It is conceivable that such interplay between particles and the surrounding biological environment may lead to autoreactivity against self-epitopes, resulting in a persistent cell-mediated immune response. Further studies are needed to confirm this hypothesis.

The transient expansion of NK cells, at Day 8, further supports that the innate immune activation triggered by the NP exposure might represent an early event in the activation of CD4^+^ T-cells. Our data demonstrate that the majority of NK cells express high density of the NKR-P1A receptor indicating a predominantly T-cell-activating function rather than an inhibitory role since it was previously shown that only NK cells with low expression of this receptor inhibit T-cell proliferation (Kheradmand et al., 2008). We detected signs of IL-2 and IFN-y expression both in airways and serum 2 days post-expo sure, indicating that TiO_2_ exposure may trigger T-cell proliferation and bias toward a T_H_1 immune response already at early time points. This finding is in contrast with a recently reported study where nano-TiO_2_ particles induced a T_H_2 cell response in mice (Park et al., 2009; Larsen et al., 2010). The discrepancy is likely explained by species differences in initiation of immune responses in addition to TiO_2_ particle differences. We also observed signs of T-cells with regulatory function, as indicated by the elevated numbers of CD4^+^ T-cells expressing high surface density of the CD25 receptor. This T-cell population might thus play a role in regulating the inflammatory response, although more specific markers for regulatory T-cells, such as FoxP3, are needed for a clear-cut functional definition. Another explanation to the T-cell activation could be genetically determined. Lorentzen and colleagues demonstrated in the DA rat that chronic inflammatory joint diseases induced by adjuvants are genetically determined by variations in C-type lectin receptors (Lorentzen et al., 2007). These receptors are preferentially expressed on dendritic cells, neutrophils, macrophages, and B-cells, and are implicated in antigen recognition and uptake, cellular adhesion, signal trans-duction, and T-cell costimulation (Cambi and Figdor, 2003; Geijtenbeek et al., 2004). It is possible that this pathway of immune activation also plays a role in triggering T-cell activation in our model of NP exposure.

Notably, a tendency toward a biphasic expression pattern of IL-1α, IL-6, IFN-γ, and TNF-α was observed in serum yielding a second response 16 days post-exposure in conjunction with the peak expression of neutrophils and lymphocytes in airways. Thus, it is likely that the long-term effects triggered by nano-TiO_2_ particles are not limited to a local cellular response in the lungs, but also includes a systemic production of cytokines stimulating T_H_1 inflammatory responses. Our findings of decreased serum levels of VEGF levels from Days 8 to 90 post-exposure might indicate a protective mechanism aimed at limiting endothelial permeability in lung inflammation. Maitre and colleagues reported a similar decrease in serum VEGF in a bacteria-induced lung injury model (Maitre et al., 2001).

## Conclusions

We demonstrated that a single high-dose exposure of TiO_2_ NP into the lung may provoke long-lasting lymphocyte responses in the DA rat, having implications on the assessment of risks for adverse and persistent immune stimulation in susceptible individuals. We also demonstrated NP-induced immunoactivating and proinflammatory activity in blood, implicating the risk for cardiovascular toxicity of inhaled NPs. Acknowledgements

We thank Christine Akfur, Christian Lejon, Barbro Ekström-Hammarström, Camilla Österlund, Mona Koch, and Lina Ågren for technical assistance. We thank Dr. Sofia Jonasson for critical reading of the manuscript.
